# Evaluation of Physics‐Based Protein Design Methods for Predicting Single Residue Effects on Peptide Binding Specificities

**DOI:** 10.1002/jcc.70160

**Published:** 2025-06-26

**Authors:** Merve Ayyildiz, Jakob Noske, Florian J. Gisdon, Josef P. Kynast, Birte Höcker

**Affiliations:** ^1^ Department of Biochemistry University of Bayreuth Bayreuth Germany

**Keywords:** armadillo repeat protein, BBK*, binding affinity calculation, binding pocket design, flex ddG, PocketOptimizer, protein–peptide interactions

## Abstract

Understanding the interactions that make up protein–protein or protein‐peptide interfaces is a crucial step towards applications in biotechnology. The mutation of a single residue can have a strong impact on binding affinity and specificity, which is difficult to capture in sampling and scoring. Many established computational methods provide an estimate of binding or non‐binding; however, comparing highly similar ligands is an important and significantly more challenging problem. Here we evaluated the capability of predicting ligand binding specificity using three established but conceptually different physics‐based methods for protein design. As a model system, we analyzed the binding of peptides to designed armadillo repeat proteins, where a single residue of the peptide was changed systematically, leading to affinity changes in the range of 1–1000 nM. We critically assessed the prediction accuracy of the computational methods. While a good correlation with experimentally determined data was observed in several cases, specific biases in the prediction performance of each method were identified.

## Graphical abstract

The ability to predict protein interactions with high precision is immensely useful. Here we tested different computational methods that use physics‐based force fields on an experimental dataset and evaluated how well these predictions can be used to discriminate binding pockets on a single residue level.

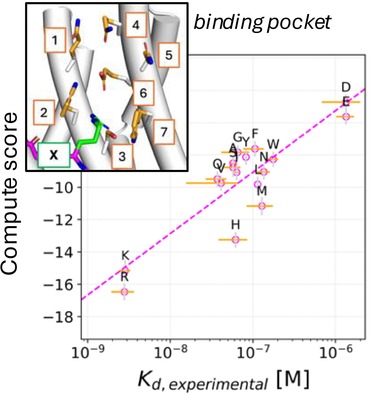

## 1 Introduction

The function of proteins often relies on the specific recognition of target molecules such as chemical compounds, peptides, or other proteins. This specific recognition is facilitated by a combination of individual interactions at the binding interface. Due to the diverse nature of interactions between amino acids, it is challenging to predict the effect of single mutations on the interaction with the target. Affinity towards one target can be reduced or even lost upon mutations at an interface [1, 2]. Specificity, however, does not require maintaining the same magnitude of affinity, as long as one target is predominantly recognized over other potential binding partners [3, 4].

Evaluating single residue effects on binding specificity requires representative structures or structural ensembles, which can be scored by a scoring function that estimates different energetic contributions to the binding [5, 6]. Machine‐learning based scoring functions have been developed for the approximation of binding specificity [7, 8], however, they often do not include routines for preparing mutated structural models or ensembles of models. In addition, single residue resolution is still a challenge even for large structure prediction models [9]. Furthermore, while point mutation effects have been analyzed for protein‐peptide systems, these analyses did not include mutations to all natural amino acids, especially in a very close range of *K*_D_ values [10, 11, 12, 13]. These factors and the less straightforward interpretability are reasons that no machine learning model has been as broadly applied as the physics‐based methods [14, 15].

In this work, we evaluated predictions of protein‐peptide binding specificities using three conceptually different physics‐based approaches. First, we investigated the flex ddG approach [16] from the widely used Rosetta software suite, which calculates binding affinity changes upon mutation. By keeping non‐mutated regions fixed, contributions from less critical components of the interaction are reduced, thereby simplifying the calculations, although this approach is not ideal as it may neglect important contributions. With the backrub approach [17, 18], a diverse ensemble is generated that is structurally close to the initial complex and is used in the calculation. Second, we applied the Branch and Bound Over K* (BBK*) approach [19] from the Osprey software suite [20]. In contrast to K*, BBK* is an accelerated algorithm that bounds multisequence partition functions, used to optimize over large sequence spaces. It is based on the approximation of the partition functions for the bound and unbound states of a system, the protein‐ligand complex and the free protein and ligand, respectively. The calculated K* scores for a protein‐ligand complex approximate the binding affinity constant *K*_a_ and are defined as the quotient of the partition functions [20]. Third, we included the PocketOptimizer approach [20], which generates an ensemble of the bound state and determines the energetically best combination of side chain rotamers and the ligand position in the binding pocket.

**FIGURE 1:**
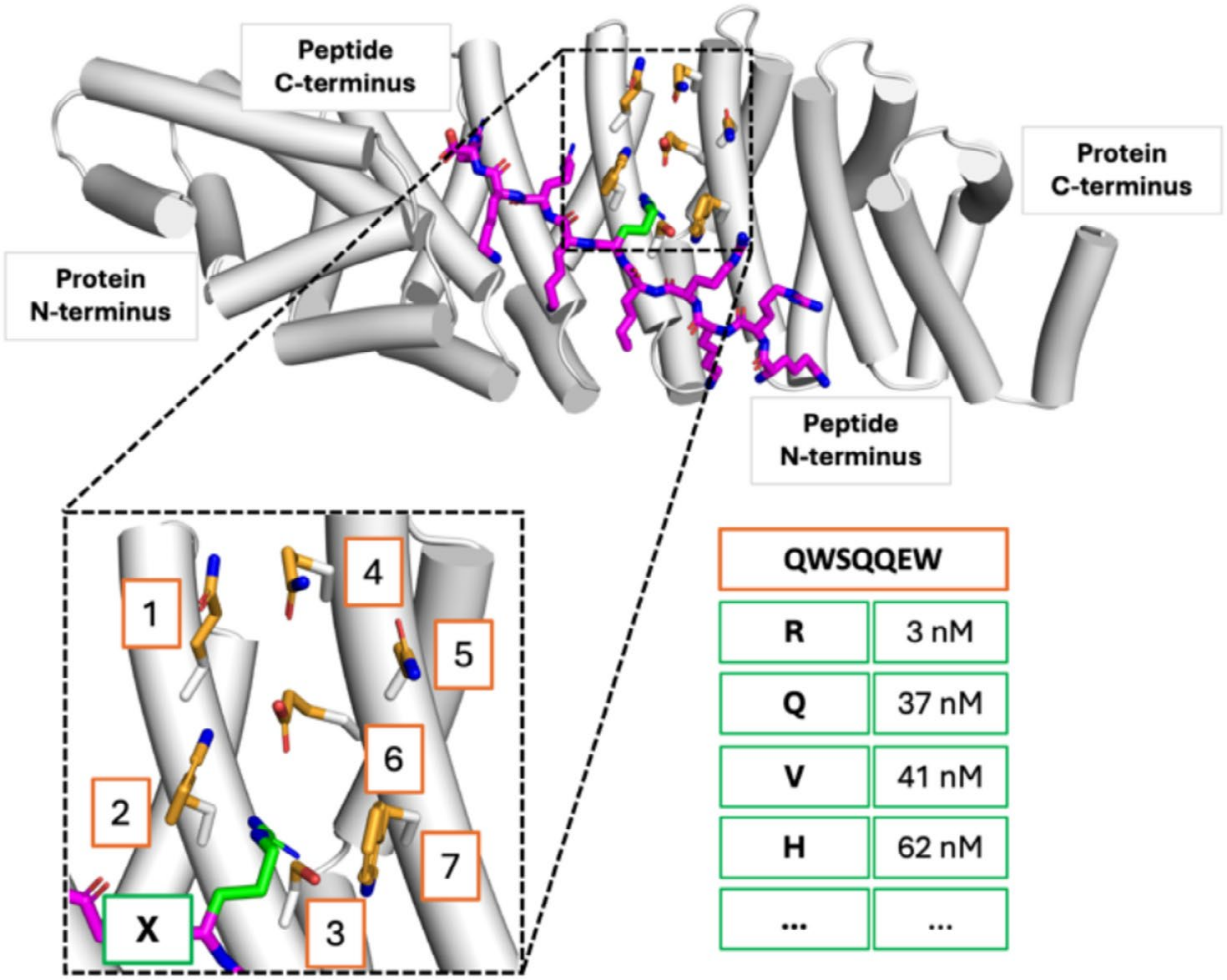
Model system for the computation of single residue effects on peptide binding specificities. The crystal structure of a designed armadillo repeat protein (dArmRP) is shown as white cartoon (PDB ID: 6SA8). A peptide consisting of five alternating Lys and Arg residues ((KR)_5_) that binds to the dArmRP is shown as magenta sticks. The designed binding pocket consisting of seven residues (orange sticks) is shown in the enlarged inset. The side chain of the Arg residue (labeled X) is shown in green. The table illustrates experimental binding affinities for peptides with a mutation of the amino acid that binds to the designed binding pocket (residue identities in the orange box).

As a model system for our study, we chose a designed armadillo repeat protein (dArmRP) with a peptide ligand that is mutated at one distinct position (Figure 1). This system has been targeted by protein engineering to develop a modular system for specific peptide recognition [21, 22]. The availability of experimental data with an affinity range from 1 to 1000 nM for many single residue variants allows us to focus on a small variable interaction region [22]. We evaluated the relative binding affinities of a set of five diverse, experimentally characterized binding pockets (see Table S1) and compared the results with each other. The characteristics and biases of all three methods are illustrated, highlighting what can be expected from available physics‐based methods.

## 2 Results and Discussion

We initially applied the three algorithms flex ddG, BBK*, and PocketOptimizer to score protein‐peptide complexes where the peptide ligand is mutated at a single position relative to the respective binding pocket of the designed armadillo repeat protein (dArmRP) (Figure 1). By that, we obtained predictions for the binding of all protein‐peptide pairs for which experimental binding data was available to evaluate the specificity of the addressed binding pockets. This allowed us to correlate the calculated binding specificities with the experimentally determined ones to assess the quality of the predictions.

### 2.1 Comparison of Binding Specificity Predictions With Experimentally Determined Data

**FIGURE 2:**
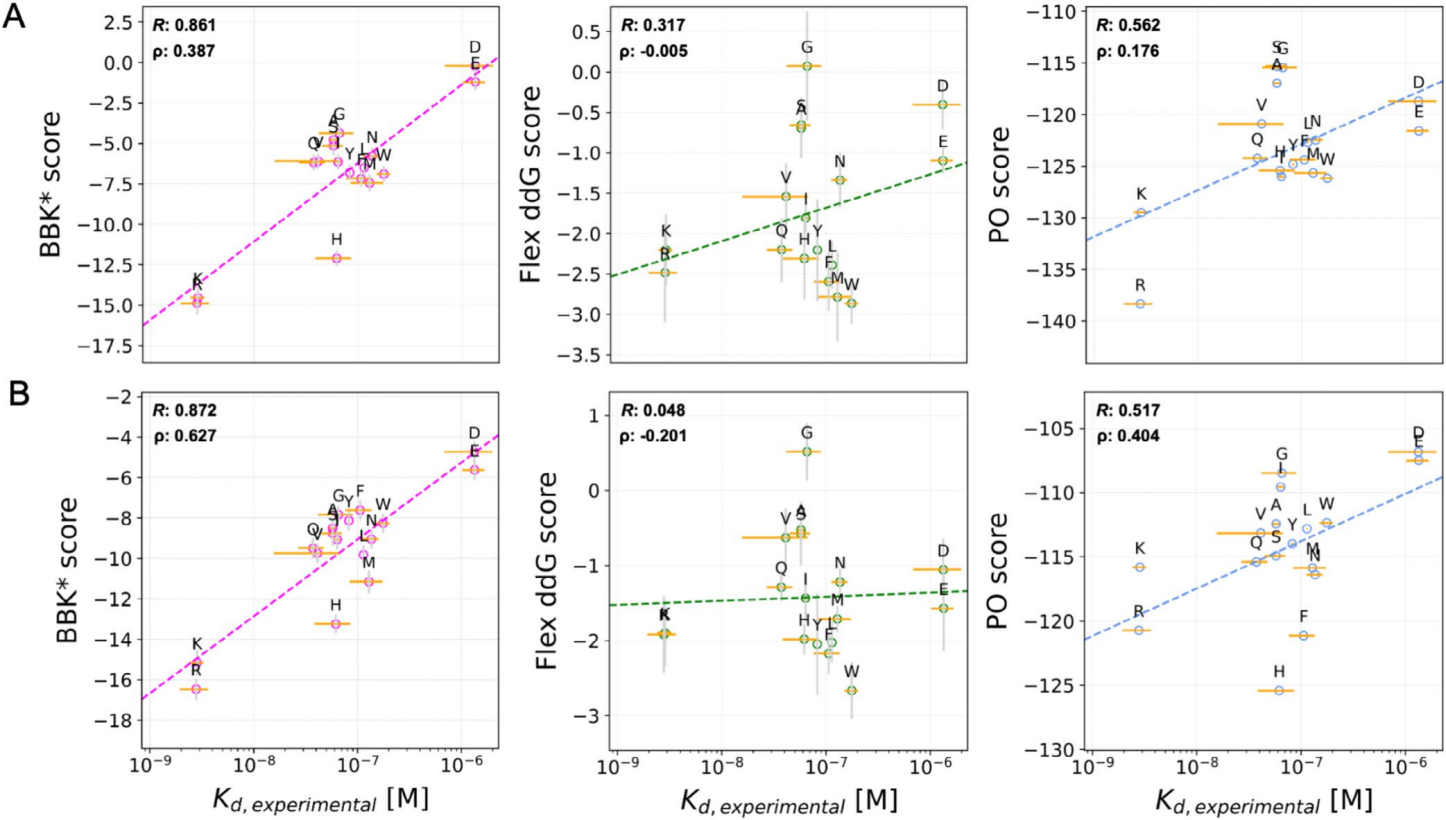
Correlation of calculated and experimentally determined binding specificities. Specificity predictions compared to experimental data for the Arg‐binder pocket (Linear fits shown in dashed lines). Binding specificity predictions from BBK* (magenta), flex ddG (green) and PocketOptimizer (blue) were correlated with experimentally determined binding specificities using the crystal structures 6SA8 (A) and 5AEI (B) as scaffold. Pearson correlations are given inside the corresponding plots. BBK* scores represent pK_D_ values, flex ddG scores affinity changes in kcal·mol^−1^, and PO scores binding energies in kcal·mol^−1^.

In a first step, we compared the binding specificities calculated using flex ddG, BBK*, and PocketOptimizer with the experimentally determined binding affinities of the so‐called Arg‐binder (see Figure 1). This was done for calculations based on two different available crystal structures, namely PDB‐ID 6SA8 and 5AEI (Figure S1), thereby gaining insights into the dependency of the predictions on the input structure (Figure 2A,B).

The Arg binding pocket has shown high affinity in experiments for the positively charged amino acids Arg and Lys [23]. The *K*_D_ for these residues is in the low nanomolar range, whereas the *K*_D_ for peptides with the negatively charged amino acids Asp or Glu at the same position is in the micromolar range (Figure 2, *K*_D, *experimental*_). The measurements of peptides with every other proteogenic amino acid at this position are clustered in between with similar *K*_D_ values. The partially overlapping error margins of this middle group add to the similarity and make the individual targets hard to distinguish. Thus, the specificity prediction of BBK* reflected well the experimentally measured trend with a Pearson R of over 0.86 (Figure 2, left). The calculated specificities allowed to distinguish between the ligands with positively and negatively charged amino acids at the variable position. Ligands with uncharged amino acids showed similar predicted affinities and clustered in between the ligands with positively and negatively charged amino acids at the variable position. Only the partially positive amino acid His was predicted to bind with higher affinity compared to the experimental values.

With flex ddG on the other hand, ligands with positively and negatively charged amino acids were distinguished well only when based on the structure 6SA8 (Figure 2A, center), while the difference between these two amino acid groups was not resolved for 5AEI (Figure 2B, center). This is also reflected in the Pearson R, which decreases from 0.317 to 0.048. Small amino acids like Gly, Ala, and Ser were predicted to be overall worse than most other amino acids. All other amino acids cluster in the middle with a trend indicative of a bias for large amino acids.

The predictions with PocketOptimizer were similar for both scaffolds, though the amino acids Phe and His were emphasized more in the case of 5AEI. Overall, PocketOptimizer distinguished well between the negatively and positively charged amino acids and even separated Arg from Lys. The calculations based on 6SA8 resulted in overall lower energy scores compared to the scores for 5AEI, with negatively charged residues being represented better. This leads to a reasonably high Pearson R value of 0.54 on average. For BBK* and PocketOptimizer, it should be noted that although the Pearson R values are comparable in the case of both scaffolds, the individual ranking of amino acids is better in scaffold 5AEI.

**FIGURE 3:**
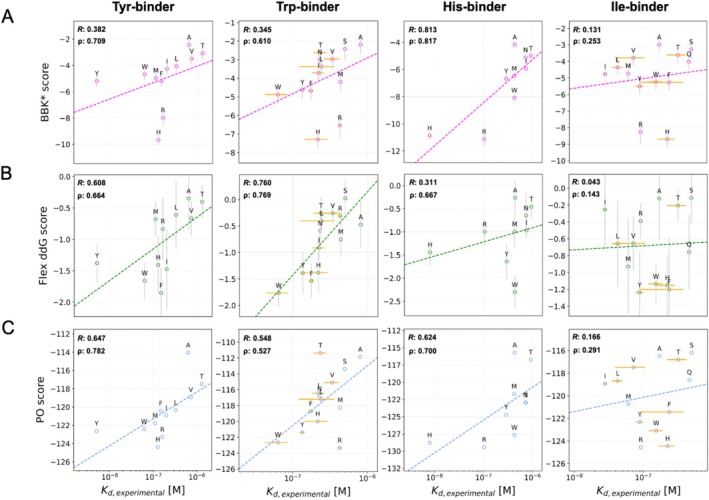
Correlation between specificity predictions and experimental binding data for Tyr, Trp, His, and Ile binding pockets. Correlation between experimental measurements for each binder with the calculations from BBK* (A), flex ddG (B), and PocketOptimizer (C) using 6SA8 as the scaffold. All predictions are shown with their corresponding Pearson correlations.

After analyzing a pocket for a positively charged amino acid, we looked at binding pockets for the aromatic residues Tyr and Trp (Figure 3, first two columns). With a *K*_D_ in the low nanomolar range and about one order of magnitude difference to other measured amino acids, the Tyr binding pocket shows a high affinity and a high specificity for Tyr (Figure 3, *K*_D, *experimental*_). Similarly, the Trp binding pocket shows a tight binding with an experimentally determined *K*_D_ in the nanomolar range and a high specificity towards Trp. Additionally, we analyzed His and Ile binding pockets (Figure 3). Based on the experimental results, the His binding pocket is well able to distinguish His from other amino acids and binds His with a high affinity. The closest amino acid target for this binding pocket is Arg with a *K*_D_ of more than one order of magnitude higher. The binding pocket Ile also shows high affinity for its target; however, the distinction of different amino acids in this position is not as clear since many of the experimentally determined error margins overlap (Figure 3, Column 4).

The specificity predictions of BBK* for the binding pockets of the aromatic amino acids Tyr and Trp did again recreate the trend observed in the experimental results. Both Tyr and Trp were ranked well for their respective pockets by BBK* (Spearman Rho: 0.709 and 0.610), with the exceptions of His and Arg, which were strongly favored. Both binding pockets were also well predicted by flex ddG. For the Trp‐binder, all calculated amino acids showed a good correlation with the experimental values (Pearson R: 0.760). The predictions for the His pocket by BBK* also correlated with the experimental values with a Pearson R of 0.813. In this case, His was the second‐best prediction for its pocket, with a slight difference from Arg, and both amino acids were predicted to be significantly better than all other amino acids. In the flex ddG predictions, the correlation is not as clear. Although flex ddG can capture the trend of the experimental results for His binding (Spearman Rho: 0.667), the amino acids Tyr and Trp were rated with higher affinity indicating a bias for these aromatic amino acids. The specificity calculations by PocketOptimizer, on the other hand, showed consistently good predictions for Tyr, Trp, and His binding pockets (Pearson R: 0.647, 0.548 and 0.624); there was, however, a bias observed towards Arg and His in each case.

In contrast to these generally good correlations, all three methods had difficulties predicting the experimentally observed trends for the Ile binding pocket. While BBK* and PocketOptimizer ranked Ile in the middle of the other amino acids (Spearman Rho: 0.253 and 0.291), it is ranked as one of the worst binding ones by the flex ddG method (Spearman Rho: 0.143) (Figure 3, Column 4). For all test cases, Ile was a difficult target to predict as it is not a large or charged amino acid. This also made the Ile binding pocket a more challenging test case. In addition, the experimental values determined for the Ile‐binder are closer to each other than for any other binders making the case even more tricky.

Overall, repeating the predictions, which were based on the 5AEI structure, with the 6SA8 structure showed comparable trends (Figure S2). Small differences might be due to the architecture of the constructs. In 6SA8, the peptide‐binding interface is protected from crystal contacts by a fused DARPin protein, while the binding interface in the 5AEI structure is not protected and is involved in some crystal contacts (Figure S1). However, the DARPin fusion might also affect the bending of the armadillo repeat superhelix, which could lead to slight changes in the prediction results.

### 2.2 Prediction Method Biases

**FIGURE 4:**
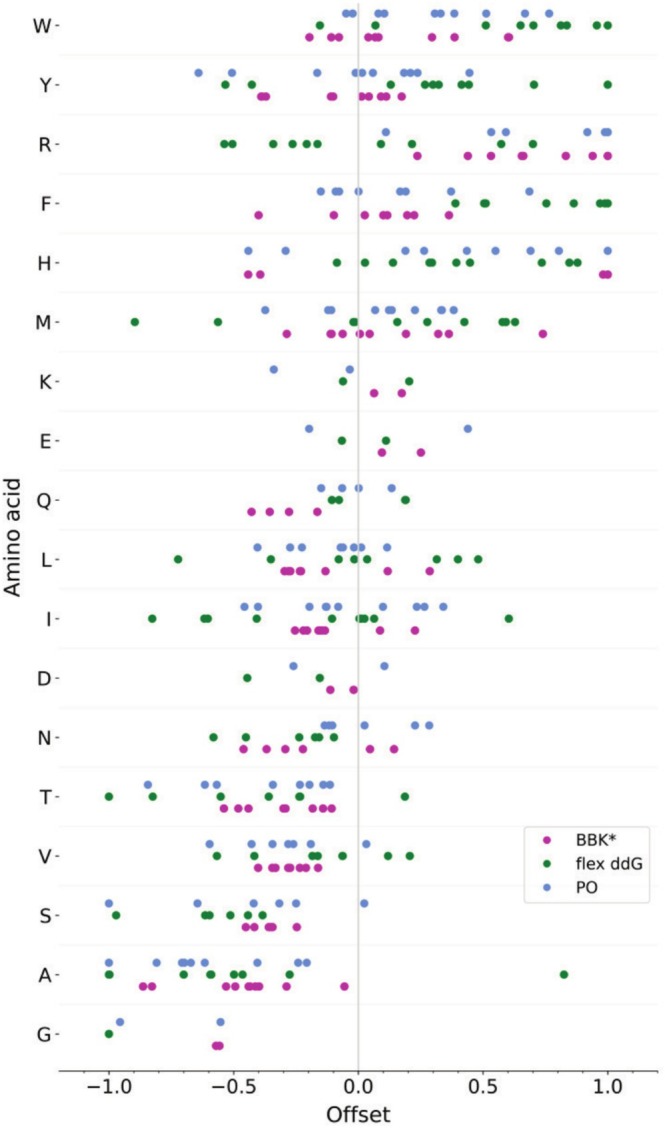
Individual relative offsets from optimal fit for individual amino acid targets. Amino acids are listed on the *y*‐axis according to their relative mass.

There are known tendencies towards overpredicting energetic contributions of certain amino acids [24, 25]. We observed similar biases in our predictions. To address individual tendencies of the methods towards different amino acid types, we calculated a relative bias for each target peptide with the corresponding amino acid (Figure 4). A trend was observed towards an overemphasis of larger amino acids, which could be explained by a general tendency of rotamer sampling for overpacking the binding pocket and/or limitations of the underlying scoring functions. Prediction offsets for Tyr were lower on average, but the individual offsets were spread throughout the offset range. This might be due to Tyr having a high affinity to most of the examined binding pockets, also the calculation of the offsets was less accurate for the edge cases. For charged amino acids, which are medium to large, the prediction offset was low, especially for Lys, Glu and Asp. However, these three amino acids lack experimental validation for several pockets making the data basis rather small. Further, Arg is highly overemphasized by BBK* and PocketOptimizer. In comparison the flex ddG predictions seemed more balanced. Despite these trends the calculation of relative offsets needs to be interpreted with caution; due to the normalization of the offset, its absolute error might differ drastically for different pockets.

### 2.3 Correlations Between Binding Specificity Predictions From BBK*, Flex ddG, and PocketOptimizer

**FIGURE 5:**
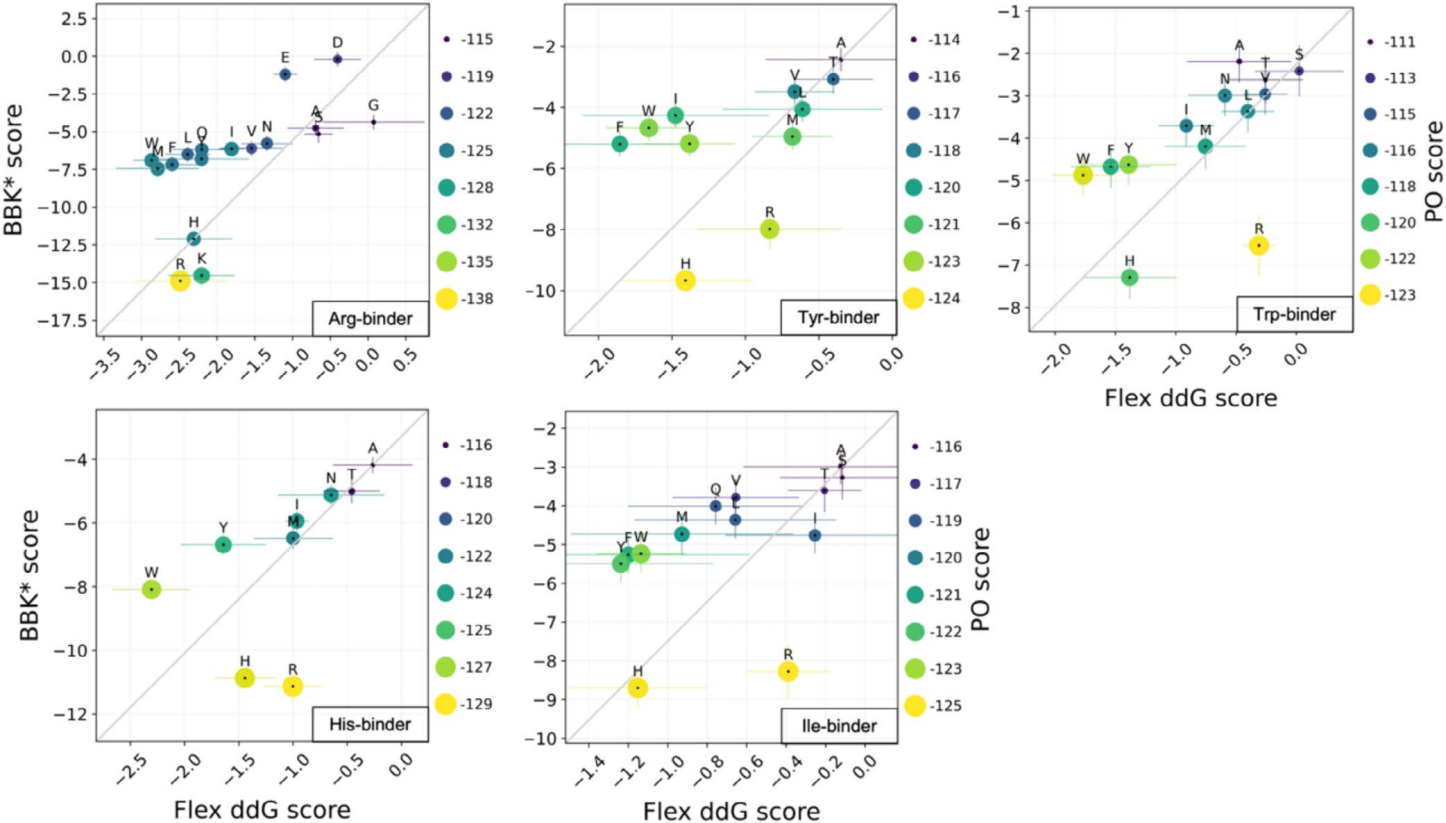
Correlation of specificity predictions from all three methods. BBK*, flex ddG, and PocketOptimizer predictions for Arg, Tyr, Trp, His, and Ile binders were obtained using the crystal structure 6SA8 as the scaffold. Flex ddG and BBK* values are shown on the *x* and *y*‐axis, respectively, while PocketOptimizer values are shown as color and size of the data points, with the values written on the right side of each plot. Error bars are from flex ddG and BBK* calculations.

We correlated the methods BBK*, flex ddG, and PocketOptimizer with each other considering the five binders analyzed above (Figure 5). The aim was to analyze the strengths that each method has and increase the accuracy of specificity predictions by combining results from different methods. For the Arg‐binder, BBK* and PocketOptimizer values correlated well with each other. For the Tyr‐ and Trp‐binders flex ddG performed better and corrected His and Arg tendencies that the other two methods show. His was ranked well in the His‐binder by all methods. In contrast, the Ile‐binder was not predicted well by any of them. Therefore, the correlation was less informative for these two binders. A similar overall trend could also be seen for predictions based on scaffold 5AEI (Figure S3). We believe that the correlations can be helpful by providing robustness of the predictions. Nonetheless, as we pointed out previously all three methods share a similar bias towards overpredicting larger amino acids, and this bias can therefore not be expected to be reduced by a complementary analysis.

## 3 Conclusion

Peptide‐binding pockets that can recognize diverse target amino acids were evaluated with binding specificity calculations using three conceptually different physics‐based methods. BBK* from Osprey as well as PocketOptimizer rely on fully physics‐based force fields whereas flex ddG from Rosetta also includes empirical terms for its energy function. We compared the specificities of single amino acid binding pockets for different residue types and evaluated the prediction accuracy of the three approaches. Flex ddG seemed to more accurately predict the specificity of binding pockets for aromatic amino acids, while BBK* performed better on pockets for positively charged amino acids. PocketOptimizer on the other hand showed a lower, but more consistent Pearson correlation in those test cases. These shortcomings can to some extent be accounted for by a complementary analysis using all three methods in combination. This way, individual biases can be corrected by another prediction method. However, in cases where the methods share similar biases an orthogonal method that reflects other aspects of the binding would be immensely useful. For example, entropic contributions might play a larger role in peptide binding, especially in the armadillo repeat proteins where the peptide is bound in an extended conformation [26].

With this evaluation, we provide a detailed overview of the prediction accuracy of widely used physics‐based protein design software for binding specificity. Our analysis is performed in a unique dataset that includes many single residue variants. We believe that the biases of the prediction methods we observed and that persisted even when different scaffolds were used are inherent to the methods and valuable information for general users. This analysis makes apparent what can be expected from available physics‐based methods. It further highlights that single residue predictions are still a great challenge and need to be addressed, in particular with respect to rational protein design and therapeutic peptide development. Maybe this can be tackled by new approaches, such as machine learning‐based algorithms or advanced sampling methods. The analysis and benchmark provided here will be a valuable instrument to test and evaluate their prediction accuracy.

## 4 Materials and Methods

### 4.1 Preparation of Structural Models

dArmRPs generated through consensus design [27] were chosen to serve as scaffold proteins since they selectively bind extended peptides in a modular fashion. The proteins bind to a repetitive, positively charged sequence, namely a (KR)_5_ peptide. All simulations were based on the two crystallographic structures with PDB‐ID 6SA8 and 5AEI that were both solved in complex with the (KR)_5_ ligand. The crystal structure 6SA8 entails a dArmRP‐fusion with a designed ankyrin repeat protein (DARPin) that arranges in a ring‐like structure [28] while 5AEI includes only the dArmRP [23]. The structures were prepared for the simulations with MoleculeKit [29] as follows: all ions, water molecules, and crystallization additives were removed. For the models based on 6SA8, the protein residues 179 to 514 of chain A were used so that the DARPin fusion was excluded. Chain B with residues 1–10 was used as ligand. For the models based on 5AEI, the protein residues 11–291 of chain A were taken. The residues 1–10 of chain D were used as ligand.

For each structure, 5AEI and 6SA8, five models were prepared according to experimentally characterized protein sequences for an Arg‐binder, a Tyr‐binder, a Trp‐binder, a His‐binder, and an Ile‐binder [22]. All mutations were introduced with PyMOL [30] and 2000 cycles of molecular sculpting were performed, which is a procedure within PyMOL to return local atomic geometries to the initial configuration of the crystal structure. Seven residues in the binding pockets of the model proteins were mutated according to experimentally determined sequences [22]. The respective residue numbers for models based on 6SA8 are 364, 368, 371, 403, 406, 407, and 410, while for models based on 5AEI, they are 155, 159, 162, 194, 197, 198, and 201.

In the 6SA8 structure, residue 6 of the peptide ligand binds to the prepared binding pocket. For the calculations with BBK* and flex ddG, residue 6 for the input structure was modeled as alanine and was allowed to mutate to all other amino acids within the respective method. For the calculations with PocketOptimizer, all input structures with the respective amino acid at position 6 of the ligand were generated. In accordance with the experimental setup, residue 4 and 8 of the ligand were modeled as alanines. In 5AEI, the respective residue of the peptide ligand is residue 4 (Figure S1).

Protonation states were determined with PropKa 3.2 [31]. Uncertain protonation states of amino acids, which were predicted to titrate less than 0.5 pH units from the adjusted pH 7, were manually adjusted according to present interactions.

### 4.2 Computational Evaluation of Binding Affinity Scores

For the evaluation of binding affinities, we used three different methods: (1) BBK* in Osprey 3.2.304 [19], (2) flex ddG in Rosetta 3.12 [16], and (3) PocketOptimizer 2.0 [32].

1. For the calculations with the BBK* algorithm, we applied side chain flexibility to all seven residues, which specify the binding pocket (Figure 1). Four of the seven residues were modeled with continuous flexibility, which is the option to rotate continuously within a range of ±9° from the discrete values of the rotamer library [33]. For models that are based on 6SA8, discrete flexibility was applied to residues 364, 403, and 406, and continuous flexibility to residues 368, 371, 407, and 410. For the ligand, residue 6 was modeled with continuous flexibility. Backbone flexibility was applied to ligand residues 2–8. For the models that are based on 5AEI, discrete flexibility was applied to residues 155, 194, and 197, and continuous flexibility to residues 159, 162, 198, and 201. For the ligand, residue 4 was modeled with continuous flexibility. Backbone flexibility was applied to ligand residues 2–8. For the calculations, we used the implemented Amber96 forcefield with the EEF1 solvation model [34]. We applied an epsilon of 0.68 as suggested to be sufficient to distinguish between orders of magnitudes of K*.
2. For the simulations with the flex ddG algorithm, we used the same protocol as described in [16]. For each model, we generated ensembles with 250 structures. A sufficient ensemble size for robust results was determined with initial tests. For each generated structure, the backrub algorithm [18] was run for 35 000 backrub Monte Carlo steps. A snapshot structure was stored every 5000 steps, resulting in seven structures. We used the Talaris all‐atom energy function [35]. The score analysis was performed as described, using the corresponding reweighting scheme based on a generalized additive model [16].
3. The program PocketOptimizer 2.0 [32] was used with the Amber ff14SB force field [36] to calculate all interaction energies between flexible side chains, ligand poses, and the fixed scaffold structure. Since the dArmRP is highly charged, the electrostatic component was scaled down to 1% based on initial tests. Minimization of side chains was carried out by applying a constant force of 5 kcal·mol^−1^·Å^−2^ to all heavy backbone atoms, and the energy was minimized until convergence. Rotamer sampling for all pocket positions was carried out, selecting rotamers from the Dunbrack backbone‐dependent rotamer library with a probability of 1% or above [37]. For the mutable ligand position, the C.M. Lib backbone‐independent rotamer library [38] was used. All sampled peptide conformations were translated by ±0.1 Å and rotated by ±2.5° along each axis. Of the rotamers and peptide poses sampled, those with a vdW energy of more than 50 kcal mol^−1^ in a scaffold where all pocket positions are mutated to an alanine were pruned before the energy calculations.

### 4.3 Processing and Analysis of Calculated Binding Affinity Scores

The output data of BBK* and flex ddG was further processed. For our analysis, we restricted the comparison of the predicted scores to the available experimental data (Table S1). The obtained K* scores from BBK* were converted into approximate p*K*_D_ values. As an uncertainty range, the obtained upper and lower bounds of the K* score were taken. The obtained flex ddG scores represented binding affinity changes in kcal mol^−1^ relative to the respective alanine references. We considered the best 5% of the calculated values from the simulated ensemble and calculated the mean value with the mean absolute error to represent the average score for the energetic change in the binding energy. In PocketOptimizer the scores represent the binding energy in kcal mol^−1^ between the protein and the peptide. Since PocketOptimizer selects one combination of pocket rotamers and a single ligand pose that represent the global minimum energy conformation (GMEC) in the sampled set of conformations, there is only a single energy value without error computed for each complex. The Pearson correlation coefficient R was used to assess the linear relationship between the computationally predicted and experimentally determined binding affinity values. The calculation was performed using the following formula:

$$R=\frac{\sum \left(X_{i}-\bar{X}\right)\left(Y_{i}-\bar{Y}\right)}{\sqrt{\sum {\left(X_{i}-\bar{X}\right)}^{2}\;\sum {\left(Y_{i}-\bar{Y}\right)}^{2}}}$$

*X*_*i*_ and *Y*_*i*_ represent individual data points in the two datasets *X* and *Y*, respectively. $\bar{X}\;\text{and}\;\bar{Y}$ are the mean values of *X* and *Y*.

Additionally, the Spearman's rank correlation coefficient (*ρ*) was used to evaluate the strength and direction of the monotonic relationship between the predicted and experimental binding affinity values. Spearman's rho is based on the ranked values of the data rather than the raw data itself. The calculation was performed using the following formula:

$$\rho =1-\frac{6\sum d_{i}^{2}}{n\left(n^{2}-1\right)}$$

*d*_*i*_ is the difference between the rank of the corresponding values of *X*_*i*_ and *Y*_*i*_, and *n* is the total number of data points.

### 4.4 Calculation of Prediction Biases

For each method on each tested binding complex, a linear fit was calculated between the experimentally determined binding affinity and the predicted scores. The offset of the predicted score from each fit was extracted and normalized to limit the highest absolute offset for each method/pocket combination to 1. This way, we extracted individual relative offsets from an optimal fit for individual amino acid targets. We sorted the relative offsets in the order of the molecular mass of the amino acid.

## Conflicts of Interest

The authors declare no conflicts of interest.

## Supporting information


**Data S1.** Supporting Information.
**Figure S1**. Crystal structures of dArmRP proteins used in this study.
**Figure S2**. Correlation between predicted and experimentally determined binding specificities.
**Figure S3**. Correlation of calculated binding specificity predictions.
**Table S1**. Experimental binding affinity data used for comparison with calculated scores.

**Data S2.** Supporting Information including code to recreate analysis.

## Data Availability

The data that support the findings of this study are openly available and provided within this manuscript and the Supporting Information.
