# Supplementary material for: Evaluation of Physics‐Based Protein Design Methods for Predicting Single Residue Effects on Peptide Binding Specificities
Source: J Comput Chem. 2025 Jun 26;46(17):e70160. doi: 10.1002/jcc.70160 (PMC12202725; doi:10.1002/jcc.70160)
Supplement: Supplementary file 1 — Data S1. Supporting Information. Figure S1. Crystal structures of dArmRP proteins used in this study. Figure S2. Correlation between predicted and experimentally determined binding specificities. Figure S3. Correlation of calculated binding specificity predictions. Table S1. Experimental binding affinity data used for comparison with calculated scores. [file JCC-46-0-s001.pdf]

Supplementary Information for:

Evaluation of physics-based protein design methods for predicting single residue effects on peptide binding specificities

Merve Ayyildiz[1], Jakob Noske[1], Florian J. Gisdon[1], Josef P. Kynast[1], Birte Höcker

Department of Biochemistry, University of Bayreuth, 95447 Bayreuth, Germany

Birte Höcker. E-mail: birte.hoecker@uni-bayreuth.de

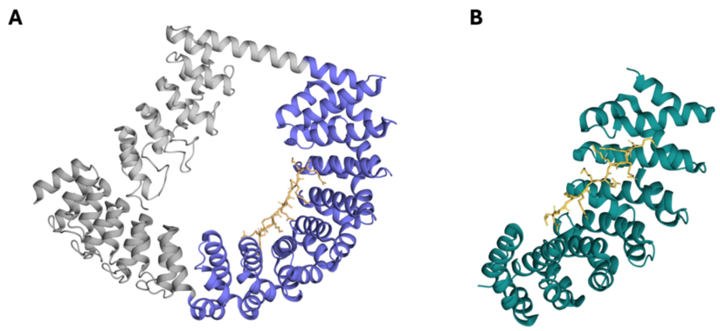

Fig S1: Crystal structures of dArmRP proteins used in this study. The structures with PDB-ID 6SA8 (A) and 5AEI (B), both with bound $(KR)_5$ peptides shown as orange sticks, were used for the calculations. The two armadillo-peptide complexes are structurally highly similar and align with an all-atom RMSD of 0.58 Å.

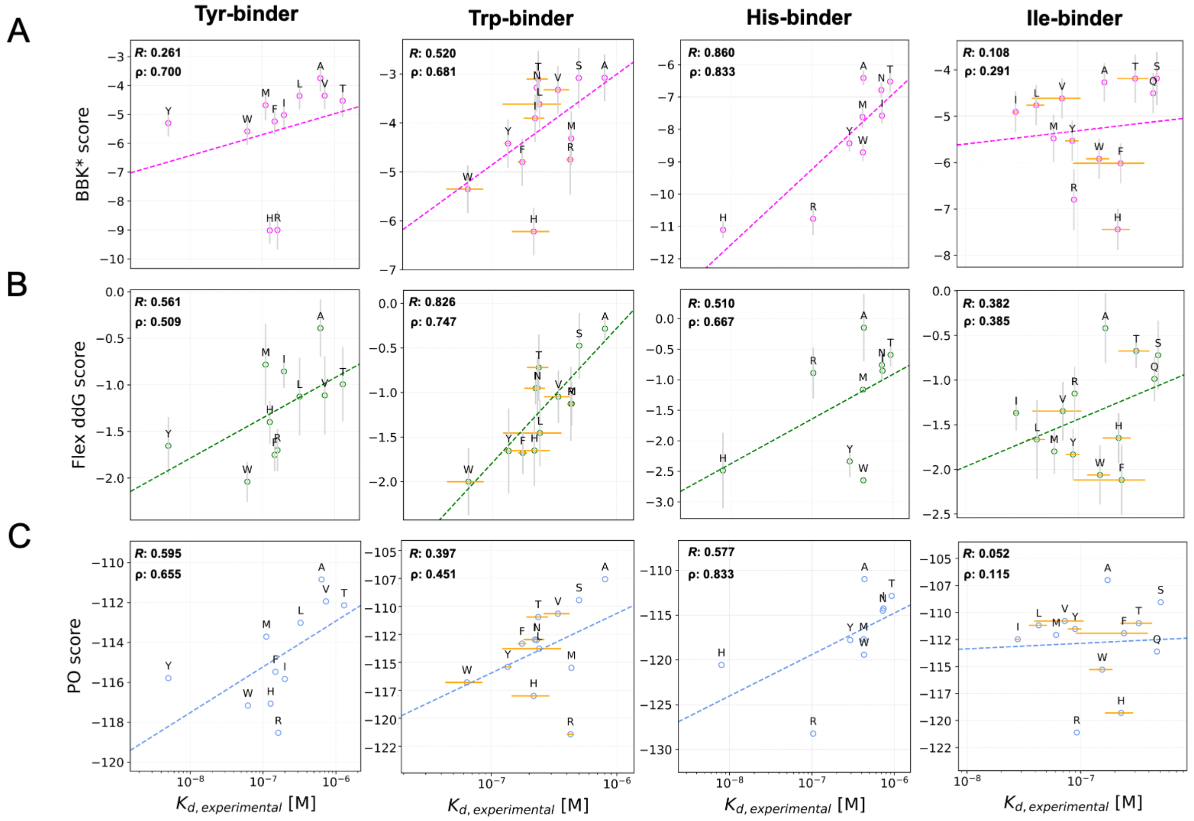

Fig S2: Correlation between predicted and experimentally determined binding specificities. Crystal structure 5AEI was used as scaffold where binding pockets for Tyr, Trp, His, and Ile were introduced.

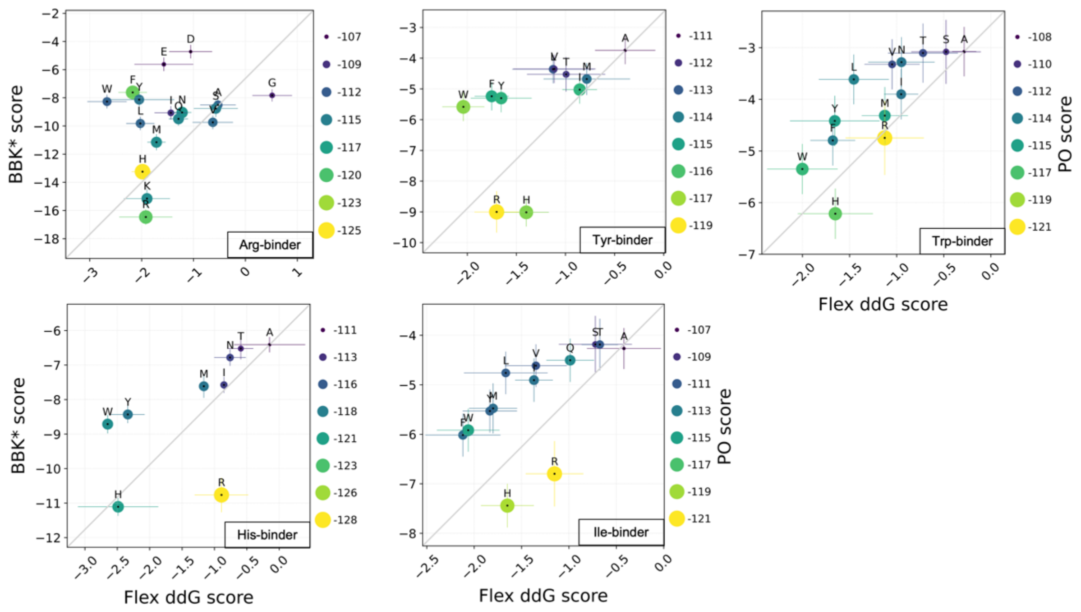

Fig S3: Correlation of calculated binding specificity predictions. Crystal structure 5AEI was used as scaffold for the introduction of Arg, Tyr, Trp, His and Ile binding pockets and the algorithms flex ddG, BBK* and PocketOptimizer were used for the specificity predictions.

Table S1: Experimental binding affinity data used for comparison with calculated scores.

**Arg-binder** (QWSQQEW)		
Peptide	K_D [nM]	Error [nM]
R	2.8	0.9
K	2.9	0.4
Q	37.3	10.4
V	41.1	25.5
A	57.6	4.8
S	57.6	13.0
H	61.8	23.5
I	63.6	4.1
G	65.7	24.2
Y	82.5	1.9
F	105.9	29.8
L	114.0	1.6
M	128.6	44.9
N	136.1	23.9
W	176.3	26.5
D	1334.0	652.0
E	1343.5	323.2

**Tyr-binder** (KEVLIRQ)		
Peptide	K_D [nM]	Error [nM]
Y	5	-
W	62	-
M	111	-
H	127	-
F	148	-
R	162	-
I	199	-
L	327	-

A	636	-
V	731	-
T	1296	-

**Trp-binder** (TATAWRT)		
Peptide	K_D [nM]	Error [nM]
W	64	21
Y	135	11
F	176	15
H	218	73
I	223	42
N	230	-
T	236	46
L	241	119
V	340	80
R	428	28
M	435	-
S	500	-
A	806	-

**Ile-binder** (FALYDRV)		
Peptide	K_D [nM]	Error [nM]
I	28	1
L	43	8
M	61	-
V	73	34
R	93	-
Y	90	12
W	155	36
A	173	-
H	227	63
F	241	150

T	325	100
Q	470	-
S	507	-

**His-binder** (DYTDWQA)		
Peptide	K_D [nM]	Error [nM]
H	8	-
R	104	-
Y	292	-
M	425	-
W	430	-
A	436	-
N	727	-
I	740	-
T	930	-
